# London Homœopathic Hospital

**Published:** 1919-12-06

**Authors:** 


### London Homœopathic Hospital.

A PRETTY POUND DAY.

THE fourth annual pound day was held on November 25 in the Board Room of the London Homœopathic Hospital at Great Ormond Street, which, for the occasion, had been decorated with flowers by the Ladies' Guild.

Lady Durning-Lawrence was greeted by the matron, and then she received the numerous gifts from those who had responded to the appeal.

The need for a successful pound day was especially felt on account of a bank overdraft exceeding £10,000, which has been incurred in meeting the high cost of all necessities during the war. The children who participated in the proceedings provided a pleasant spectacle as they arrived in class formation from the neighbouring school of St. George the Martyr, carrying their little bundles ready to place in baskets provided for the occasion.

The contributions were varied, chiefly comprising tea, cocoa, rice, and oatmeal, making a total exceeding 800 lb. in the case of household commodities, and a sum approaching £150 in money. Tea and light refreshments at 1s. per head were served by the sisters, who succeeded in raising a further £5. Music formed part of the afternoon's programme.

